# Relationship between residence characteristics and polycyclic aromatic hydrocarbon exposure in housewives: second Korean National Environmental Health Survey (2012–2014)

**DOI:** 10.1186/s40557-018-0236-x

**Published:** 2018-04-18

**Authors:** Hyung-Gue Park, Na-Young Ha, Dae Hwan Kim, Jeong-Ho Kim, Chae-Kwan Lee, Kunhyung Kim, Ji Young Ryu

**Affiliations:** 1 Department of Occupational and Environmental Medicine, Inje University Haeundae Paik Hospital, 875 Heaundae-ro, Haeundae-Gu, Busan, 48108 South Korea; 2 Department of Occupational and Environmental Medicine, Institute of Environmental and Occupational Medicine, Busan Paik Hospital, Inje University, 75, Bokji-ro, Busanjin-gu, Busan, 47392 South Korea

**Keywords:** PAHs, Housewives, Residence characteristics, Heating fuel

## Abstract

**Background:**

Polycyclic aromatic hydrocarbons (PAHs) produced by incomplete combustion have negative effects on human health due to their carcinogenicity and teratogenicity. Indoor sources of PAHs include tobacco smoke, heating sources, and cooking. This study evaluated the relationship between human PAH exposure and residence characteristics.

**Method:**

This study was based on the second Korean National Environmental Health Survey (2012–2014). Non-smoking housewives were included in the analyses (*n* = 1269). The concentrations of urinary PAH metabolites (2-naphthol, 2-hydroxyfluorene, 1-hydroxyphenanthrene, and 1-hydroxypyrene) were adjusted by urine creatinine level. The geometric mean concentrations of urinary PAH metabolites by residential factors were examined. Logistic regression models were used to evaluate associations between residential variables and PAH exposures.

**Results:**

The adjusted geometric mean concentrations of urinary 2-hydroxyfluorene and 1-hydroxyphenanthrene were significantly higher in the group residing within 100 m of a major road (*p* < 0.05) than in those residing > 100 m from a major road. In logistic regression analyses, the odds ratio (OR) for exceeding the third quartile of urinary 1-hydroxypyrene concentration was significantly higher in the group using coal or wood fuel for residential heating than in the group using gas (OR = 2.745, 95% confidence interval [CI] = 1.295–5.819). The detached house group had a significantly higher OR for 1-hydroxyphenanthrene compared with the apartment group (OR = 1.515, 95% CI = 1.023–2.243).

**Conclusion:**

Our study shows the evidence of associations between some urinary PAH metabolite levels (1-hydroxyphenanthrene and 1-hydroxypyrene) and residence characteristics. Additional studies are needed to clarify these associations.

## Background

Polycyclic aromatic hydrocarbons (PAHs) are ubiquitous environmental pollutants containing two or more fused aromatic rings. PAHs are produced by the incomplete combustion of various organic materials, including coal, oil, gas, and wood. Major emission sources of PAHs include vehicle exhaust, industrial activity, power generation, waste incineration, and residential heating [1]. Tobacco smoke, heating systems, cooking, and infiltration of outdoor pollutants contribute to indoor PAH levels [2].

Before the 2000s, most studies of PAH exposure focused on occupational exposure such as in coke oven workers, aluminum smelter workers, and road pavers [3]. Subsequently, however, related research has focused on low-level environmental PAH exposure. Owing to difficulties in exposure source identification and the demand for integrated data on human exposure through multiple routes, including inhalation, ingestion, and dermal contact, biomonitoring using urine has allowed the assessment of non-occupational PAH exposure.

There are more than 100 PAHs. The United States Environmental Protection Agency lists 16 typical PAHs as priority compounds due to health concerns [4]. Among them, the following seven PAHs are classified as probable carcinogens: benzo(a)anthracene, chrysene, benzo(b)fluoranthene, benzo(k)fluoranthene, benzo(a)pyrene, indeno(1,2,3-c,d)pyrene, and dibenzo(a,h)anthracene [5]. The International Agency for Research on Cancer (IARC) also categorizes benzo(a)pyrene, the most highly carcinogenic of these compounds, as group 1 (carcinogenic to humans). It is particularly associated with lung, bladder, and skin cancers [6]. Also, several other PAHs are classified as probably or possibly carcinogenic to humans (IARC groups 2A and 2B, respectively) [7]. In addition to their carcinogenicity, other adverse health effects, including the development of cardiovascular [8, 9] and respiratory [10, 11] diseases, may be associated with chronic PAH exposure. Moreover, epidemiological studies suggest that prenatal exposure to PAHs can cause birth defects [12, 13] and impaired neurodevelopment [14, 15].

Indoor air quality exerts a considerable impact on human health. Indeed, indoor PAH levels in air or dust are related to residence characteristics, including age and type [16–18]. However, the PAH concentration in air or dust may not directly reflect personal exposure. Few studies have investigated indoor PAH exposure using urinary PAH metabolite levels. In this study, we evaluated the relationship between residential factors and PAH exposure by determining the levels of PAH metabolites in urine.

## Methods

### Study participants

In this study, we used data from the second Korean National Environmental Health Survey (KNEHS), which was conducted by the National Institute of Environmental Research from 2012 to 2014; the survey involved stratified sampling of the national population and the 2010 housing census. The survey comprised 6478 participants from 400 districts who were selected based on the population distribution. Data were collected by means of person-to-person interviews and biological sampling. We selected 1760 subjects identified as housewives, aged > 19 years, and non-smokers. Subjects who had outlier PAH metabolite concentrations (*n* = 53) and who did not respond to the relevant questions (*n* = 438) were excluded. Finally, 1269 subjects were included in the analysis.

### Variables

Age, body mass index (BMI), second-hand smoking, alcohol drinking, and exercise were included as variables in this study. Regarding residence characteristics, type of heating fuel, type of residence, age of building, and distance from a major road were analyzed. Type of heating fuel was categorized into the following four groups: ‘gas,’ ‘oil,’ ‘coal/briquette/wood,’ and ‘electric fuel/cogeneration.’ Type of residence was divided into the following three groups: ‘apartment,’ ‘multiplex,’ and ‘detached house.’ Age of building and occupancy years were categorized as ≤10, 10–20, 20–30, and > 30 years. Distance of residence from a major road was categorized into less and more than 100 m.

### Urinary PAH metabolite levels

Four PAH metabolites were analyzed: 2-naphthol (naphthalene), 2-hydroxyfluorene (fluorene), 1-hydroxyphenanthrene (phenanthrene), and 1-hydroxypyrene (pyrene). Urinary PAH metabolite concentrations were analyzed by gas chromatography-mass spectrometry. The metabolites were hydrolyzed by ß-glucuronidase/aryl sulfatase followed by derivatization with bistrimethylsilyl trifluoroacetamide. Concentrations were calculated from calibration curves drawn by the standard addition method. The limit of detection (LOD) was 0.050 μg/L for 2-naphthol, and 0.040 μg/L for 2-hydroxyfluorene, 0.047 μg/L for 1-hydroxyphenanthrene, 0.015 μg/L for 1-hydroxypyrene. For internal quality control purposes, the target coefficient of the calibration curve was ≥0.995. Measurements below the LOD were replaced with the LOD divided by the square root of 2. All urinary PAH metabolite concentrations were adjusted to the urine creatinine concentration.

### Statistical analysis

Geometric means and 95% confidence intervals of urinary PAH metabolite levels (2-naphthol, 2-hydroxyfluorene, 1-hydroxyphenanthrene, and 1-hydroxypyrene) were calculated according to demographic characteristics. Because the distributions showed positive skewness, all metabolite concentrations were natural-log transformed before use in the analyses. Univariate comparisons according to residential parameters and demographic characteristics were performed by Student’s *t*-test and an analysis of variance. An analysis of covariance was used for comparisons of variables adjusted for age and passive smoking. The adjusted odds ratios (ORs) for exceeding the upper quartile of the concentration of each urinary PAH metabolite were calculated by multiple logistic regression analyses. SPSS (version 22 for Microsoft Windows; IBM Corp., Armonk, NY) was used in all statistical analyses.

## Results

Table 1 shows the demographic characteristics and geometric mean urinary concentrations of PAH metabolites. The concentrations of all four urinary PAH metabolites differed significantly among the age groups. The urinary concentration of 1-hydroxyphenanthrene and 1-hydroxypyrene was significantly higher in the obese group than the other body weight groups. The non-alcohol-drinking group had a significantly higher urinary concentration of 2-naphthol than the alcohol-drinking group. Urinary 2-naphthol and 1-hydroxyphenanthrene concentrations were significantly higher in the non-exercise group. The geometric mean with 95% confidence intervals, median, 10th, 25th, 75th, and 90th percentile concentrations of each urinary PAH metabolite are listed in Table 2.

**Table 1:** Demographic distributions and geometric mean concentration of urinary PAH metabolites with 95% confidence intervals in μg/g creatinine

	N (%)	2-Naphthol GM (95% CI)	2-Hydroxyfluorene GM (95% CI)	1-Hydroxyphenanthrene GM (95% CI)	1-Hydroxypyrene GM (95% CI)
Age					
20–29 yrs	30 (2.4)	2.10 (1.47–3.02)	0.23 (0.18–0.29)	0.10 (0.08–0.13)	0.18 (0.14–0.23)
30–39 yrs	229 (18.0)	2.02 (1.77–2.30)	0.26 (0.24–0.28)	0.10 (0.10–0.11)	0.16 (0.14–0.17)
40–49 yrs	208 (16.4)	2.09 (1.82–2.40)	0.25 (0.23–0.28)	0.11 (0.10–0.12)	0.17 (0.16–0.19)
50–59 yrs	284 (22.4)	2.82 (2.51–3.17)	0.31 (0.28–0.33)	0.15 (0.14–0.16)	0.21 (0.19–0.22)
60–69 yrs	313 (24.7)	2.97 (2.66–3.32)	0.30 (0.27–0.32)	0.15 (0.14–0.16)	0.22 (0.21–0.24)
≥ 70 yrs	205 (16.2)	3.39 (2.95–3.89)	0.30 (0.27–0.33)	0.16 (0.14–0.17)	0.22 (0.20–0.24)
*p*-value		< 0.001	0.002	< 0.001	< 0.001
BMI					
< 25 kg/m^2^	780 (61.5)	2.56 (2.38–2.75)	0.27 (0.26–0.29)	0.13 (0.12–0.13)	0.19 (0.18–0.20)
≥ 25 kg/m^2^	489 (38.5)	2.72 (2.48–2.98)	0.29 (0.28–0.31)	0.15 (0.14–0.16)	0.21 (0.20–0.22)
*p*-value		0.297	0.072	< 0.001	0.008
Passive smoking					
No	1111 (87.5)	2.63 (2.47–2.79)	0.28 (0.27–0.29)	0.14 (0.13–0.14)	0.20 (0.19–0.20)
Yes	158 (12.5)	2.55 (2.17–3.00)	0.29 (0.26–0.32)	0.12 (0.11–0.14)	0.18 (0.16–0.20)
*p*-value		0.740	0.797	0.071	0.181
Drinking					
No	753 (59.3)	2.75 (2.55–2.96)	0.28 (0.27–0.30)	0.14 (0.13–0.14)	0.19 (0.19–0.20)
Yes	516 (40.7)	2.44 (2.23–2.67)	0.28 (0.27–0.30)	0.13 (0.12–0.14)	0.20 (0.18–0.21)
*p*-value		0.044	0.963	0.261	0.912
Exercise					
No	596 (47.0)	2.43 (2.23–2.64)	0.27 (0.26–0.29)	0.12 (0.12–0.13)	0.19 (0.18–0.20)
Yes	673 (53.0)	2.80 (2.60–3.03)	0.29 (0.27–0.30)	0.14 (0.14–0.15)	0.20 (0.19–0.21)
*p*-value		0.012	0.147	< 0.001	0.062

**Table 2:** Geometric mean with 95% confidence intervals and selected percentile concentrations of urinary PAH metabolite (μg/g creatinine)

PAH metabolite	GM (95% CI)	10th percentile	25th percentile	Median	75th percentile	90th percentile
2-Naphthol	2.62 (2.48–2.77)	0.87	1.31	2.28	4.64	10.21
2-Hydroxyfluorene	0.28 (0.27–0.29)	0.13	0.18	0.28	0.42	0.63
1-Hydroxyphenanthrene	0.13 (0.13–0.14)	0.06	0.09	0.13	0.21	0.30
1-Hydroxypyrene	0.19 (0.19–0.20)	0.09	0.14	0.20	0.29	0.43

Table 3 presents the geometric mean and 95% confidence intervals of urinary PAH metabolite concentrations according to residence characteristics adjusted for age and passive smoking. The concentrations of 2-hydroxyfluorene and 1-hydroxyphenanthrene were significantly higher in the group with a residence < 100 m from a major road (*p* < 0.05).

**Table 3:** Adjusted geometric mean concentrations (μg/g creatinine) of urinary PAH metabolites with 95% confidence intervals in μg/g creatinine categorized by residence characteristics

Category	N (%)	2-Naphthol GM^*^ (95% CI)	2-Hydroxyfluorene GM^*^ (95% CI)	1-Hydroxyphenanthrene GM^*^ (95% CI)	1-Hydroxypyrene GM^*^ (95% CI)
Type of heating fuel					
Gas	1014 (79.9)	2.54 (2.31–2.80)	0.29 (0.27–0.31)	0.13 (0.12–0.14)	0.19 (0.17–0.20)
Oil	145 (11.4)	3.18 (2.53–4.00)	0.27 (0.23–0.31)	0.13 (0.11–0.14)	0.20 (0.17–0.23)
Coal/briquette/wood	36 (2.8)	1.82 (1.07–3.08)	0.32 (0.22–0.45)	0.17 (0.12–0.24)	0.30 (0.21–0.42)
Electric fuel/cogeneration	74 (5.8)	3.15 (2.25–4.41)	0.27 (0.21–0.33)	0.15 (0.12–0.18)	0.19 (0.15–0.24)
*p*-value		0.106	0.687	0.252	0.082
Type of residence					
Apartment	741 (58.4)	2.56 (2.29–2.86)	0.28 (0.26–0.31)	0.13 (0.12–0.14)	0.19 (0.17–0.20)
Multiplex	170 (13.4)	2.65 (2.10–3.36)	0.29 (0.25–0.34)	0.13 (0.11–0.14)	0.18 (0.15–0.21)
Detached	358 (28.2)	2.73 (2.34–3.20)	0.28 (0.26–0.32)	0.14 (0.13–0.16)	0.20 (0.18–0.23)
*p*-value		0.796	0.981	0.157	0.237
Age of building					
≤ 10 yrs	155 (12.2)	2.72 (2.18–3.39)	0.29 (0.25–0.33)	0.14 (0.12–0.16)	0.18 (0.16–0.21)
10–19 yrs	382 (30.1)	2.85 (2.43–3.36)	0.29 (0.26–0.33)	0.13 (0.12–0.15)	0.18 (0.16–0.20)
20–29 yrs	465 (36.6)	2.40 (2.09–2.74)	0.27 (0.25–0.30)	0.13 (0.12–0.14)	0.19 (0.17–0.21)
> 30 yrs	267 (21.0)	2.67 (2.20–3.24)	0.29 (0.26–0.33)	0.13 (0.12–0.15)	0.21 (0.19–0.24)
*p*-value		0.405	0.713	0.756	0.336
Distance from major road					
≥ 100 m	594 (46.8)	2.57 (2.27–2.91)	0.27 (0.25–0.29)	0.12 (0.11–0.13)	0.18 (0.17–0.20)
< 100 m	675 (53.2)	2.68 (2.38–3.00)	0.30 (0.28–0.33)	0.14 (0.13–0.15)	0.20 (0.18–0.22)
*p*-value		0.644	0.043	0.018	0.118

*Estimated geometric means and *p*-values were calculated by ANCOVA adjusting for age and passive smoking

The results of logistic regression analyses adjusted by age and passive smoking are shown in Table 4. The coal/briquette/wood fuel group had a significantly higher OR for 1-hydroxypyrene compared with the gas group (OR = 2.745, 95% confidence interval [CI] = 1.295–5.819). The OR for 1-hydroxyphenanthrene was significantly higher in the detached house group than in the apartment group (OR = 1.515, 95% CI = 1.023–2.243).

**Table 4:** Adjusted odds ratios for exceeding the third quartile of each urinary PAH metabolite by residence characteristics

Category	2-Naphthol OR^a^ (95% CI)	2-Hydroxyfluorene OR^a^ (95% CI)	1-Hydroxyphenanthrene OR^a^ (95% CI)	1-Hydroxypyrene OR^a^ (95% CI)
Type of heating fuel				
Gas	1	1	1	1
Oil	1.515 (0.979–2.343)	0.865 (0.543–1.378)	0.734 (0.458–1.175)	1.145 (0.721–1.820)
Coal/briquette/wood	0.668 (0.289–1.543)	1.504 (0.707–3.200)	1.136 (0.530–2.435)	2.745 (1.295–5.819)
Electric fuel/cogeneration	0.962 (0.530–1.745)	0.465 (0.228–0.946)	0.918 (0.507–1.660)	0.884 (0.469–1.668)
Type of residence				
Apartment	1	1	1	1
Multiplex	1.250 (0.840–1.862)	1.447 (0.984–2.128)	1.439 (0.967–2.142)	1.203 (0.808–1.793)
Detached	1.290 (0.872–1.908)	1.206 (0.809–1.796)	1.515 (1.023–2.243)	0.997 (0.664–1.497)
Age of building				
≤ 10 yrs	1	1	1	1
10–19 yrs	1.213 (0.748–1.968)	0.942 (0.590–1.505)	0.912 (0.563–1.477)	1.329 (0.807–2.189)
20–29 yrs	1.029 (0.637–1.662)	1.039 (0.657–1.642)	0.996 (0.623–1.594)	1.247 (0.762–2.043)
> 30 yrs	1.517 (0.901–2.553)	1.485 (0.895–2.465)	1.139 (0.679–1.913)	1.631 (0.951–2.798)
Distance from major road				
≥ 100 m	1	1	1	1
< 100 m	1.108 (0.848–1.447)	1.270 (0.971–1.660)	1.213 (0.925–1.590)	0.965 (0.737–1.264)

^a^Adjusted by age, passive smoking

## Discussion

In this study, we assessed the relationship between residence characteristics and PAH exposure. PAH metabolite concentrations differed significantly according to distance from a major road, and were associated with using solid fuel and living in a detached house.

When comparing with national survey data of the United States and Canada among female population, the concentrations ranges of the urinary 2-hydroxyfluorene and 1-hydroxyphenanthrene in our study population were similar with the results of these countries [19, 20]. But our results showed higher urinary concentration of 1-hydroxypyrene and lower concentration of 2-naphthol than these countries’ results. Lower urinary concentration of 2-naphthol in our study seems to be a reflection of non-smoking population because urinary concentration of naphthalene metabolites is more sensitive to smoking status than other PAH metabolites [21].

Physical properties of PAHs vary according to its ring number, that is, molecular weight. Some researchers have evaluated that the ambient concentration of indoor low-molecular-weight PAHs (containing 2–3 rings) are mainly higher than outdoor and affected by residence characteristics and indoor activities in non-smoker home [18, 22]. Another study identified that mothballs and some building materials significantly contributed to indoor naphthalene concentrations [23]. On the other hands, outdoor sources such as traffic combustion usually emitted high-molecular-weight PAHs (containing 4 or more rings) [22, 24]. Several studies showed that there were reasonably strong correlations between indoor and outdoor concentrations in high-molecular-weight PAHs [18, 22]. Outdoor air may influence on the concentration of indoor high-molecular-weight PAHs.

Several studies have assessed air PAH concentrations by type of heating fuel. In one such study, air PAH concentrations in residences with wood-burning heating appliances were high due to intrusion of outdoor air or leakage from the appliance [25]. In our study, the OR of 1-hydroxypyrene was significantly higher in residences with coal/briquette/wood heating systems. Solid fuels, including wood and coal, reportedly emit higher levels of PAHs and other pollutants than gas fuels such as liquid petroleum gas and natural gas [26]. Also, solid fuel heating system may be more prone to leakage due to deterioration. Indeed, replacement with improved stoves reportedly reduces PAH exposure [27, 28].

The OR for 1-hydroxyphenanthrene in the detached house group was significantly higher than that in the apartment group. The PAH concentrations in outdoor air are reportedly higher on lower floors [16]. Thus, the high OR in the detached house group may be due to the smaller number of floors compared to apartments. Secondly, the concentrations of semi-volatile organic compounds (e.g., PAHs and nicotine) are high in old buildings [29, 30], possibly due to dust accumulation in carpets or adsorption onto household surfaces such as painted wallboards. However, urinary PAH metabolite levels did not differ according to building age in this study.

Road traffic is the dominant contributor to indoor PAH levels in urban areas [31]. Distance from a major road is related to influx of outdoor pollutants. Indeed, air PAH concentrations are significantly higher within 1 km of a main road and expressway [22]. In our study, the adjusted concentrations of 2-hydroxyfluorene and 1-hydroxyphenanthrene were significantly higher in the group whose residences were < 100 m from a major road. However, there was no significant difference in the concentration of 1-hydroxypyrene (the highest-molecular-weight PAH analyzed in this study) according to distance from a major road.

The main strength of the present study is the analysis of a large dataset from a standardized population. Furthermore, most previous experimental and epidemiological studies used PAH concentrations in air or dust, which hampered the evaluation of direct human exposure. Biologic monitoring using urinary metabolites provides a more direct measure of the internal body load. Semi-volatile PAHs with 4–5 rings are typically present in both the gas and particulate phases [21]; Whereas the use of air or dust PAH concentrations allows only one route of exposure to be evaluated, biomonitoring provides an integrated measure of exposure through inhalation, ingestion, and dermal absorption.

Our research has several limitations. First, the KNEHS lacks information about sampling season and behavioral habits. Ambient concentrations of PAHs may have variations among seasons. The concentration of high-molecular-weight PAHs become elevated in winter due to domestic heating [22] and low-temperature combustion [32]. For low-molecular-weight PAHs, indoor concentration is significantly higher in summer because of increased PAH vaporization with increasing temperature [18]. Also, PAHs can be affected by indoor-outdoor air ventilation which is more common in the spring and fall. On the other hand, frequent dust cleaning is negatively associated with indoor PAH concentrations [33]. Cooking habit and the use of mothballs and incense may also influence indoor pollutant levels [34]. However, the KNEHS lacks data on sampling seasons and lifestyle factors such as cleaning frequency. Thus, our results should be interpreted with caution. Second, the effect of indoor smoking cannot be completely excluded. Although non-smokers were analyzed in this study and passive smoking was included as a covariate to exclude any effect of smoking, household surfaces contaminated by indoor smoking can affect indoor PAH exposure; this effect is termed third-hand smoke. Third, because of the small number of coal/briquette/wood fuel group (*N* = 37), our result should be interpreted with caution. It could be possible that a small sample size leads to a false positive result. Future studies with larger samples are needed for more reliable result.

The health risks of PAHs have been recognized as a public concern. Because people spend a lot of time in their residences, indoor environment can contribute to daily exposure to PAHs. Therefore, it is important to evaluate the residential factors that can affect indoor PAHs exposure. In this regard, our study, which used dataset from large population, will provide the reference for future study and the evidence for developing policy of improving indoor air quality.

## Conclusion

This study investigated the relationship between residence characteristics and PAH exposure using urinary biomarkers. There was an evidence of association between the 1-hydroxypyrene level and fuel type used in the heating system. Moreover, the urinary 1-hydroxyphenanthrene level was associated with building type. Future studies should evaluate the influence of heating fuel and take into consideration the seasonality of fuel use. Also, the influence of lifestyle factors (e.g., cleaning frequency) on indoor PAH levels should be assessed.

## Abbreviations

BMI: Body mass index
CI: Confidence interval
KNEHS: Korean National Environmental Health Survey
NIER: National Institute of Environmental Research
OR: Odds ratio
PAH: Polycyclic aromatic hydrocarbon
